# Multi-trait ridge regression BLUP with *de novo* GWAS improves genomic prediction for haploid induction ability of haploid inducers in maize

**DOI:** 10.3389/fpls.2025.1614457

**Published:** 2025-08-19

**Authors:** Yu-Ru Chen, Ursula K. Frei, Thomas Lübberstedt

**Affiliations:** Department of Agronomy, Iowa State University, Ames, IA, United States

**Keywords:** genomic selection, haploid inducer, multi-trait, ridge regression BLUP, *de novo* GWAS, predictive ability

## Abstract

**Introduction:**

Ridge regression BLUP (rrBLUP) is a widely used model for genomic selection. Different genomic prediction (GP) models have their own niches depending on the genetic architecture of traits and computational complexity. Haploid inducers have unique trait performances, relevant for doubled haploid (DH) technology in maize (*Zea mays L.*).

**Methods:**

We evaluated the performance of single-trait (ST) and multi-trait (MT) GP models, which include rrBLUP, BayesB, Random Forest, and xGBoost, using data from multifamily DH inducers (DHIs). We integrated multi-trait and *de novo* genome-wide association studies (GWAS) within the rrBLUP framework to model four target traits: days to flowering (DTF), haploid induction rate (HIR), plant height (PHT), and primary branch length (PBL). Predictive ability (PA) was assessed through five-fold cross-validation and further validated in multi-parent advanced generation intercross (MAGIC) DHIs.

**Results:**

The average PAs of different GP methods across traits were 0.51 to 0.69. ST/MT *de novo* GWAS rrBLUP methods increased PA of HIR. In addition, MT GP models improved PA by 12% on average across traits relative to ST GP models in MAGIC DHIs.

**Discussion:**

These findings highlight the potential benefits of integrating multi-trait modeling or *de novo* GWAS into the rrBLUP framework. Such GP approaches in this study enhance PAs and provide empirical evidence for accelerating the genetic improvement of maize haploid inducers.

## 1 Introduction

Haploid inducers are critical for doubled haploid (DH) technology in maize breeding programs (Aboobucker et al., 2022; Chaikam et al., 2019; Röber et al., 2005). They enable the production of DH lines while accelerating line development. Compared to using the conventional pedigree method in corn breeding, a population of completely homozygous lines were obtained after two seasons rather than at least six seasons by the pedigree method. Continuous genetic improvement of inducers is needed to enhance the efficiency of DH technology and adaptation to different environments and regions of the world.

Plant breeding programs culling inferior progenies in samples of segregating populations are shifting from evaluating the phenotypes of all lines in the field to predicting the breeding values of individuals without phenotyping in early generations (Cooper et al., 2014). Meuwissen et al. (2001) proposed genomic selection (GS) to determine genomic estimated breeding values (GEBVs) of individuals without phenotyping. GEBVs are the sum of estimated additive effects of high-density genomic markers. Genomic selection requires a training set of genotypes and phenotypes, and a genomic prediction (GP) model. Training sets consist of a set of genotyped individuals related to breeding populations of interest, which are phenotypically evaluated in a sample of environments representing the target population of environments. GP models are built by capturing the effects of quantitative trait loci (QTLs), which are in linkage disequilibrium (LD) with genome-wide single nucleotide polymorphism (SNP) markers in the training set (Jannink et al., 2010). Once a GP model has been established, it can be used to screen individuals based on their GEBVs for the trait, without the need for phenotyping in early generations (Heffner et al., 2011). This is driven by low-cost marker assays, which are cheaper than phenotyping in replicated field trials. In later generations, a decreasing number of high-performing lines are evaluated in a large number of field environments in terms of years and locations, sampled from the target population of environments.

In numerous GS studies in plants and animals, it has been shown that the reliability of GS depends on the genetic architecture and heritability of traits, LD of QTLs, molecular marker density, training population size, and the genetic relationship between the test set of genotypes (breeding population) and the training set (Hayes et al., 2009; Zhong et al., 2009; Luan et al., 2009; Daetwyler et al., 2010a; De Roos et al., 2009). Different approaches have been established to develop GP models. Parametric methods are used to estimate random marker effects for prediction. Those include ridge regression best linear unbiased prediction (rrBLUP), which is equivalent to genomic relationship BLUP (GBLUP) (Habier et al., 2007; Zhong et al., 2019). Bayesian-based methods such as BayesA, BayesB, BayesCπ, and Bayesian LASSO have different prior densities of marker effects (Meuwissen et al., 2001; Kizilkaya et al., 2010; de los Campos et al., 2009). Non-parametric methods include various methods of machine learning, which are not constrained by linear models for estimating genetic effects, such as Random Forest, xGBoost, and neural networks. All methods have achieved promising prediction results for genetic improvement in breeding (Azodi et al., 2019; Crossa et al., 2017; Li et al., 2018).

Genomic regions associated with the variability of agronomic traits have been identified by genome-wide association studies (GWASs) in major crop species (Tibbs Cortes et al., 2021). Unlike GS, association mapping is used to identify potential alleles that may affect traits of interest. GWASs often have an insufficient resolution for discovering causal genes due to extensive LD. However, markers detected by GWASs in the training set (*de novo* GWASs) can be regarded as fixed factors in the rrBLUP model for increasing prediction accuracy (Akbarzadeh et al., 2021; Chen et al., 2023; Spindel et al., 2016). Nevertheless, based on the simulation of hundreds of different genetic architectures of traits, the prediction accuracy of the rrBLUP model incorporating the peak signals of markers varied on a trait-by-trait basis. Thus, this type of GP model should be inspected before implementing it in GS (Rice and Lipka, 2019).

Numerous studies have shown promising outcomes for the application of GS to single traits. However, lines in breeding programs are usually evaluated for multiple traits. Simulation and empirical studies have revealed that implementing multi-trait response variables in GP models may have a higher prediction ability than single-trait GP models by leveraging estimates of covariance among genetically correlated traits (Bhatta et al., 2020; Cappa et al., 2022; Gill et al., 2021; Jia and Jannink, 2012; Shahi et al., 2022). The benefits of using multi-trait models are more pronounced for traits with low heritability and phenotypic traits that require costly measurements. Multi-trait models can help to reduce the training set size required for the targeted traits (Gaire et al., 2022; Guo et al., 2014).

A robust inducer line in DH technology needs an adequate haploid induction rate (HIR), appropriate flowering time, plant height, and tassel size for haploid induction (Trentin et al., 2020). The primary trait of inducers, HIR, is a quantitative trait controlled by a few major QTLs along with minor QTLs (Barret et al., 2008; Prigge et al., 2012). Two major genes were identified by map-based gene cloning. For instance, *mtl/zmpla1/nld* was located in the region of *qhir1* on chromosome 1 (Gilles et al., 2017; Kelliher et al., 2017; Liu et al., 2017) and *zmdmp* in the region of *qhir8* on chromosome 9 (Zhong et al., 2019). *zmdmp* can enhance HIR two- to threefold when mtl/zmpla1/nld is present, while *zmdmp* has a very low HIR (~0.15%) on its own (Zhong et al., 2019). Additional genes were shown to affect HIR by CRISPR–Cas9-mediated mutations. The *ZmPLD3* gene was also found to have a synergistic effect with the *mtl* gene (Li et al., 2021). The *ZmPOD65* gene is involved in reactive oxygen species bursts, influencing haploid induction (Jiang et al., 2022). Meanwhile, *C1-I* haploid inducers have been developed for efficient doubled haploid inducer (DHI) line development (Chen et al., 2024). While DHIs can be produced routinely at a large scale, HIR measurements to evaluate novel inducers require testcrossing with female donor plants and subsequent labor-intensive identification of kernels carrying embryos with only the genome of maternal donor plants. It is, therefore, difficult to complete the phenotyping of a large number of DHIs because of the laborious and time-consuming process of obtaining HIR estimates for novel inducers.

In order to enhance the genetic improvement of inducers by GS, the objectives of this study were to i) compare the predictive ability of the traits of interest using rrBLUP, BayesB, Random Forest, and xGBoost methods of GP models in a multifamily sample of DHI lines; ii) evaluate the improvement of predictive ability by employing multi-trait and *de novo* GWAS approaches in the GP model; and iii) evaluate the GP model performance in the test set of multi-parent advanced generation inter-cross (MAGIC) DH lines.

## 2 Materials and methods

### 2.1 Plant materials and experimental design

**Figure 1:**
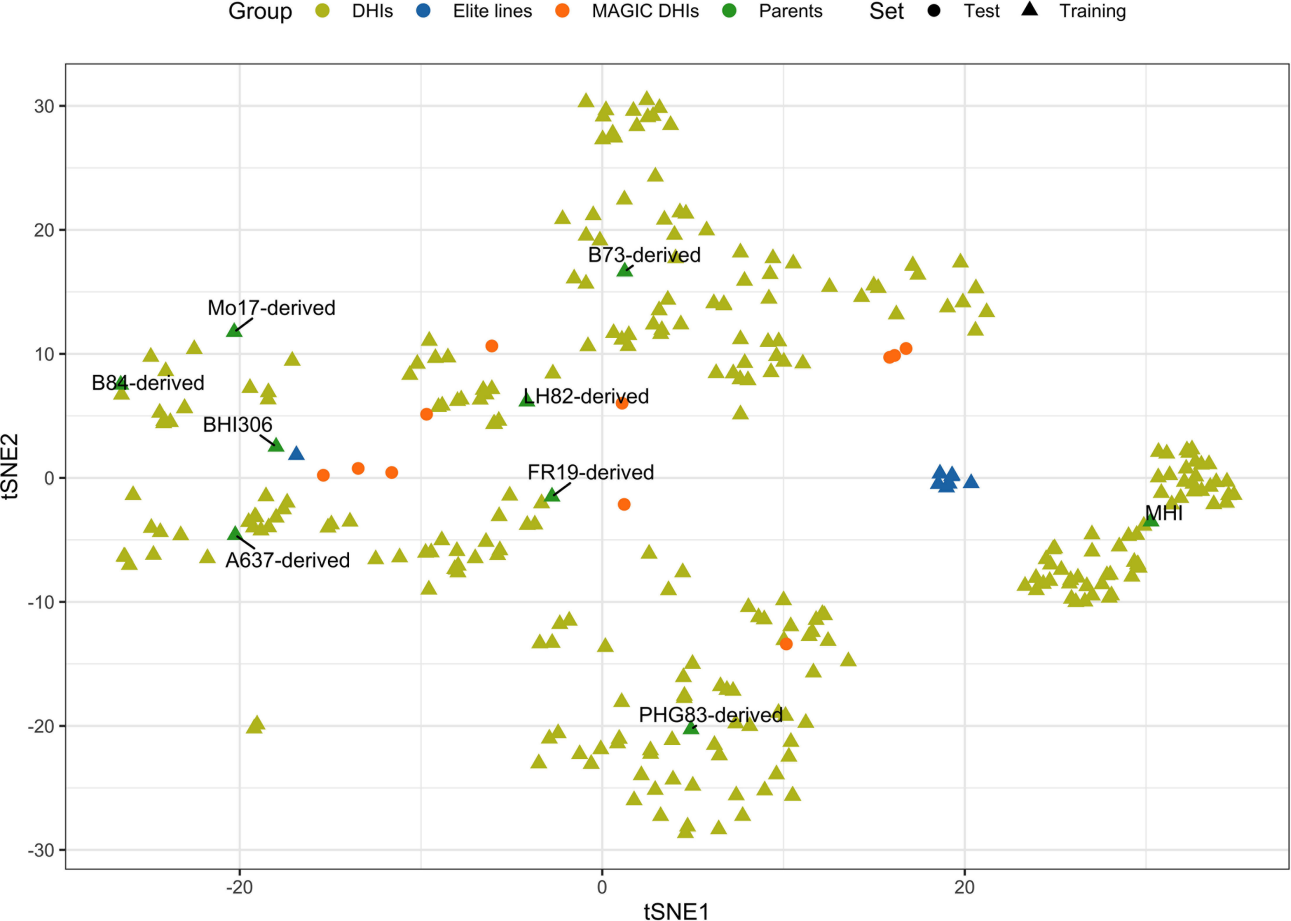
Scatterplot of genome-wide SNP genotypes of the haploid inducer lines in training and test sets, which is visualized on the first and second dimensions of t-distributed stochastic neighbor embedding (tSNE1 and tSNE2). t-SNE is a method to visualize high-dimensional data.

The 21 families used in this study consisted of DH progenies (**Supplementary Table 1**) from eight elite inducers. The eight elite inducers that were selected for the *mtl* and *zmdmp* genes were from crosses between RWS/RWK76 and A637, B73, B84, FR19, LH82, Mo17, or PHG83 non-inducer exPVP (expired Plant Variety Protection Act certificates)/public inbred lines (Chen et al., 2024). The other two families involved a traditional inducer, MHI, carrying the *mtl* gene but not the *zmdmp* gene (Zhong et al., 2019). A total of 167 and 193 entries were planted in 2021 and 2022, respectively, in 3.8-m-long field plots arranged in a randomized block design with two planting blocks at the Iowa State University Agricultural Engineering and Agronomy Farm in Boone, IA, USA. It was a complete block design for days to flowering (DTF), HIR, and plant height (PHT) but an incomplete block design for primary branch length (PBL) since the additional 231 DHI genotypes were planted in the second planting block for both seed increase and tassel measurements in 2021. All trials were sown in loamy soil under rainfed conditions, adopting standard agronomic practices for research at Iowa State University. Due to high labor intensity and limited resources for phenotypic measurements, genotypes were evaluated in an unbalanced design across the two years of the experiment. A total of 12, 11, 12, and 42 DHI genotypes were planted in both 2021 and 2022 and evaluated for DTF, HIR, PHT, and PBL, respectively, along with nine inducer parents. The eight inducer parents fixed for *mtl* and *zmdmp* were also used as founders to produce MAGIC lines. After the eight-way intercross, DH technology was applied to obtain MAGIC DHIs. Eleven MAGIC DHIs and eight other elite inducer inbreds were evaluated for their phenotypes in 2022, which were selected from the RWS/PHI-3 cross (Trentin et al., 2023). MHI was the parent of PHI-3 (Rotarenco et al., 2010). The multi-family DHIs derived from 23 families, along with the nine parents and eight elite inbreds derived from RWS/PHI-3, were regarded as the training set (**Figure 1**). The training set was also used in our GWHAS. The eleven MAGIC DHIs were used as the test set, which were unseen by GP models during the modeling process (**Supplementary Table 1**).

### 2.2 Phenotyping

The four traits of interest in inducer line development were DTF, HIR, PHT, and PBL of the tassel. DTF was measured as 50% of plants per plot shedding pollen. PHT was measured as distance (cm) from the ground to the flag leaf’s base after pollen had been shed. PBL was used to represent the tassel size of inducers, which was measured as the cumulative length (cm) of all primary branches of tassels. A commercial F_1_ hybrid, Viking 60-01N (released by Lee Seed Company, MN, USA), was used as the donor for testing the HIRs of genotypes. HIR was calculated as the number of haploid kernels divided by the total number of seeds from at least five donor cobs induced by the inducer genotypes, which were harvested after the physiological maturity (R6) stage. Suspicious haploid kernels were cut open with a scalpel to reveal whether the embryo was colorless underneath the pericarp. Entries with fewer than 800 kernels for HIR evaluation were excluded to ensure high HIR data quality (Chaikam et al., 2018)

Data analyses were conducted in R (R Core Team, 2022). The year-by-genotype interactions were significant for DTF, but not for HIR, PHT, and PBL in this 2-year dataset (**Supplementary Table 2**). DTF usually displayed the heterogeneity of genotypic variances among years in the same environment (no rank changes). For simplicity, a genotype-by-year interaction term was not included in the model. This assumed that the four traits shared a common error variance across years. The best linear unbiased estimator (BLUE) values of the trait performances were used as the adjusted phenotypic response variables of genotypes, which were estimated using the model in Equation 1:


(1) $${\text{y}}_{ijk}=\mu +{\text{year}}_{i}+\text{block}{\left(\text{year}\right)}_{j(i)}+{\text{genotype}}_{k}+\epsilon_{ijk},$$

where ɛ*ijk* is the response for genotype *k* grown in block *j* nested in year *i*, μ is the overall mean, year_i_ is the fixed effect for year, block(year)_j(i)_ is the fixed effect for block nested in years, genotype_k_ is the fixed effect of genotype, and ϵ_jk_ is the residual, which is a random variable that is independent and identically distributed as N(0, σ_ϵ_^2^). The BLUE values of genotypes estimated by the emmeans package (Searle et al., 1980) represent response variables in GP models. The reliability (*i* ^2^) on an entry mean basis was used to quantify the proportion of genetic and non-genetic effects among genotypes from phenotypic measurements, as follows:


(2) i2=σgenetic2σgenetic2+σnon−genetic2r

$\sigma_{genetic}^{2}$ and $\sigma_{non-genetic}^{2}$ were the random term of genotypes and residual term in the linear mixed model, which were estimated using the lmer package (Bates et al., 2015). r in Equation 2 is the harmonic mean of replicates of genotypes (Holland et al., 2010).

### 2.3 SNP genotyping

Seeds of DHI lines were sown in the Agronomy Greenhouse at Iowa State University. Leaf tissue from three plants per entry was harvested and lyophilized for 24 hours, and leaf samples were shipped to CIMMYT at El Batán, Texcoco, Mexico, for DNA extraction and SNP genotyping using DArTseq technology (Jaccoud et al., 2001). A total of 88,421 unimputed SNPs per line were successfully called and reported. Allele sequences were blasted against the B73 reference genome (B73 RefGen_v4) to obtain unique SNP positions and generate the HapMap files. SNP HapMap files were transformed to VCF files and numeric genotype files in TASSEL version 5.0 (Bradbury et al., 2007). Missing SNP genotypes were imputed using the default parameters of the Beagle 5.4 software (Browning et al., 2018). SNPs with minor allele frequencies less than 5% were removed. In total, 6,636 SNPs across the genome were used as feature variables in the GP models. The genotypic values of SNPs were coded as (−1, 0, 1), where 1 represents homozygosity for the major allele at a given bi-allelic locus, −1 indicates homozygosity for the minor allele, and 0 indicates heterozygous (uncertain) genotypes after the imputation of missing genotypes.

### 2.4 Genome-wide SNP association studies

The genome-wide association analysis was conducted with the GAPIT package (Version 3) (Wang and Zhang, 2021) using three models: mixed linear model (MLM) (Yu et al., 2005), fixed and random model circulating probability unification (FarmCPU) (Liu et al., 2016), and Bayesian information and linkage disequilibrium iteratively nested keyway (BLINK) (Huang et al., 2019). The coordinates on the first three principal component axes were used as the covariate variables in the models to control the population structure. The Bonferroni threshold used to detect significant SNPs associated with traits was −log(0.05/n), where n is the number of SNPs used in the association analysis. The whole set of detected SNP markers (Whole SNP) or common SNP markers detected across the three models (Shared SNP) were regarded as fixed factors in the ST/MT_GWAS_rrBLUP models, which were described in Section 2.5. The percentage of total phenotypic variance explained (PVE) by significantly associated markers was evaluated. The percentage explained by the markers was calculated as their corresponding variance divided by the total variance, which is the sum of residual variance and the variance of the associated markers.

### 2.5 Genomic prediction models

The GP models include single-trait and multi-trait approaches, which were implemented in ridge regression, mixed linear, Bayesian linear regression, and tree-based algorithmic machine learning models.

In the single-trait and multi-trait approaches of the linear model, predictions were obtained by estimating the genome-wide SNP effects using an rrBLUP model conducted using the rrBLUP package in R (Endelman, 2011) and a Bayesian B (BayesB) model with 1,000 burn-in and 6,000 iterations for the Gibbs sampler algorithm implemented in the BGLR package in R (Pérez and De Los Campos, 2014).

The single-trait linear model was defined as in Equation 3:


(3) y= μ+Xb+Zu+ϵ

where y is the vector of BLUE values for a single trait, μ is the vector of the overall mean, X is an incidence matrix of GWAS-detected SNP genotypes of individuals, and b is a vector of estimated SNP fixed effects. If no SNP was detected by a GWAS, Xb does not exist. Z is an incidence matrix of the genome-wide SNP genotypes of individuals, u is a vector of random SNP effects with $u\sim N\left(0,\;I\sigma_{m}^{2}\right)$ where I is the identity matrix, and $\sigma_{m}^{2}$ is the SNP marker variance. This approach assumes that all markers have a common variance and thus shrinks each marker effect equally toward zero in the ridge regression BLUP model, while markers have unique variances, and a proportion of them have no effect in the BayesB model (Meuwissen et al., 2001). Lastly, ϵ is the residual error vector with $\epsilon \sim N\left(0,\;I\sigma_{e}^{2}\right)$ .

The multi-trait linear models are calculated in Equations 4 and Equations 5:


(4) $$Y=\;UDV^{T}=\mu +X\beta +Zu+e$$

(5) $$Y^{*}=\mu +X\beta^{*}+Zu^{*}+e^{*}$$

where U, D , and $V^{T}$ are the matrices from the singular value decomposition (SVD) of a matrix_(n,t)_ of the responses in all traits, and Y . $V^{T}$ is an orthogonal matrix ( $V^{T}V=I$ ). Equation 5 is the transformation of Equation 4 being multiplied with V , so $Y^{*}=UD=YV$ , $\beta^{*}=\beta V$ , $u^{*}=uV$ , and $e^{*}=eV$ . $Y^{*}$ is a matrix_(n,t)_ in which the corresponding vector of trait was used as the response variable, X is an incidence matrix_(n,g1)_ of the GWAS-detected SNP genotypes of individuals, $\beta^{*}$ is a matrix_(g1,t)_ of GWAS-detected SNP marker estimates of all traits, Z is a incidence matrix_(n,g2)_ of the genome-wide SNP genotypes of individuals, $u^{*}$ is a matrix_(g2,t)_ of genome-wide SNP marker predictors of all traits, and $e^{*}$ is a matrix_(n,t)_ of residuals for all traits. n, t, g1, and g2 are the number of genotypes, traits, GWAS-detected SNPs, and genome-wide SNPs in the model, respectively. $\sum_{u}=VD_{u}V^{T}$ and $R_{e}=VD_{e}V^{T}$ , where $D_{u}$ and $D_{e}$ are diagonal variance-covariance matrices (t,t). $u^{*}$ is distributed as matrix variate distribution as $NM\left(0,\;\sum_{u}⊗\;I\right)$ and $e^{*}\sim NM\left(0,\;\;R_{e}⊗\;I\right)$ . The parameters and predicted genotypic values are approximated as $\hat{\beta }={\hat{\beta }}^{*}V^{T}$ , $\hat{u}={\hat{u}}^{*}V^{T}$ , and $\hat{Y}=X\hat{\beta }+Z\hat{u}$ (Montesinos-López et al., 2018).

The machine learning algorithmic models used in this study were Random Forest and xGBoost methods, in which the functions were from the tidymodels packages in R (Kuhn and Wickham, 2020). The BLUE values of a single trait as the target values of individuals and genome-wide SNP genotypes as the feature values of individuals were fitted in the models. The number of ensemble decision trees for regression (trees), the number of features sampled at each split (mtry), and the minimum number of individuals in a node (min_n) are the hyperparameters in Random Forest. The number of trees, the maximum depth of a decision tree (tree_depth), and the step size to update the weights of algorithmic models (learning_rate) are the hyperparameters of the xGBoost method. Mean square error was used as the loss function in the hyperparameter tuning by Bayesian optimization from the control_bayes function in the tune package in R to obtain the optimal hyperparameters in a fivefold cross-validation procedure.

### 2.6 Cross-validation

The performance of GP models was evaluated by fivefold cross-validation (CV). The training sizes of GP models for each trait are summarized in **Table 1**. The genotypic and phenotypic data of entries in the training set were shuffled and divided into equal folds of five: four used as training folds and the remaining as a held-out fold. The SNP marker effects estimated by the GP models from the training set and predicted genotypic values (GV) of individuals in the held-out fold, which are the sum of grand mean, fixed effects (if present in the models), and GEBVs (sum of random effects), were predicted. Each fivefold CV was independently repeated five times for the comparisons of GP models by means of predictive ability. In the CV procedure, the genome-wide SNP association analysis was conducted only in the four training folds in each of the five splits for the evaluation of the *de novo* GWAS rrBLUP model performance. The predictive ability (PA) is Pearson's correlation coefficient between the observed BLUE values and predicted genotypic values of the traits of the hold-out fold and test set, respectively.

**Table 1:** Summary statistics of the traits and numbers of lines used in the experiments.

Traits	Statistics					Number of genotypes		
	$\sigma_{genetypic}^{2}$	$\sigma_{non-genetypic}^{2}$	R	*i* ^2^	PVE (%)	Phenotypic measurements	Single-trait GP	Multi-trait GP
DTF	5.27	1.78	1.49	0.81	29.96	334	310	300
HIR	12.18	7.25	1.44	0.71	18.24	325	306	300
PHT	241.0	93.2	1.48	0.79	27.62	338	312	300
PBL	2379	1661	1.29	0.65	24.17	537	474	300

The number of genotypes used for phenotypic measurements and single-trait GP differed due to the failure of SNP genotyping for some DHIs. The 300 genotypes for which all four traits were determined were used in the training population of multi-trait GP. The BLUE values in the training population of multi-trait GP were used to estimate PVE. r and *i* ^2^ were the harmonic mean of replicates and the entry mean reliability for each trait, respectively.

DTF, days to flowering; HIR, haploid induction rate; PHT, plant height; PBL, primary branch length; PVE, proportion of the variance explained by the principal component of each trait; GWAS, genome-wide association study; GP, genomic prediction; DHIs, doubled haploid inducers; BLUE, best linear unbiased estimator.

### 2.7 Statistical analysis

For the visualization of the high-dimensional genotypic data of inducers in the genomic prediction, t-distributed stochastic neighbor embedding (t-SNE) was conducted in the Rtesne package in R (Krijthe, 2015). Pearson’s correlation coefficients (r) of the BLUE values among traits were calculated and visualized using the GGally package in R (Schloerke et al., 2022). The principal component (PC) analysis of the four traits was conducted using the prcomp function with the scale argument TRUE in R. The variance contributions and directions of the traits in the first two PCs were summarized using the factoextra package in R. In order to compare the differences in the PAs of GP models, the least significant difference (LSD) test of the differences among models was conducted using the agricolae package in R (Mendiburu and Yaseen, 2020) at an alpha level of 0.05.

## 3 Results

### 3.1 Summary of genotypes and phenotypes in the DHI population

**Figure 2:**
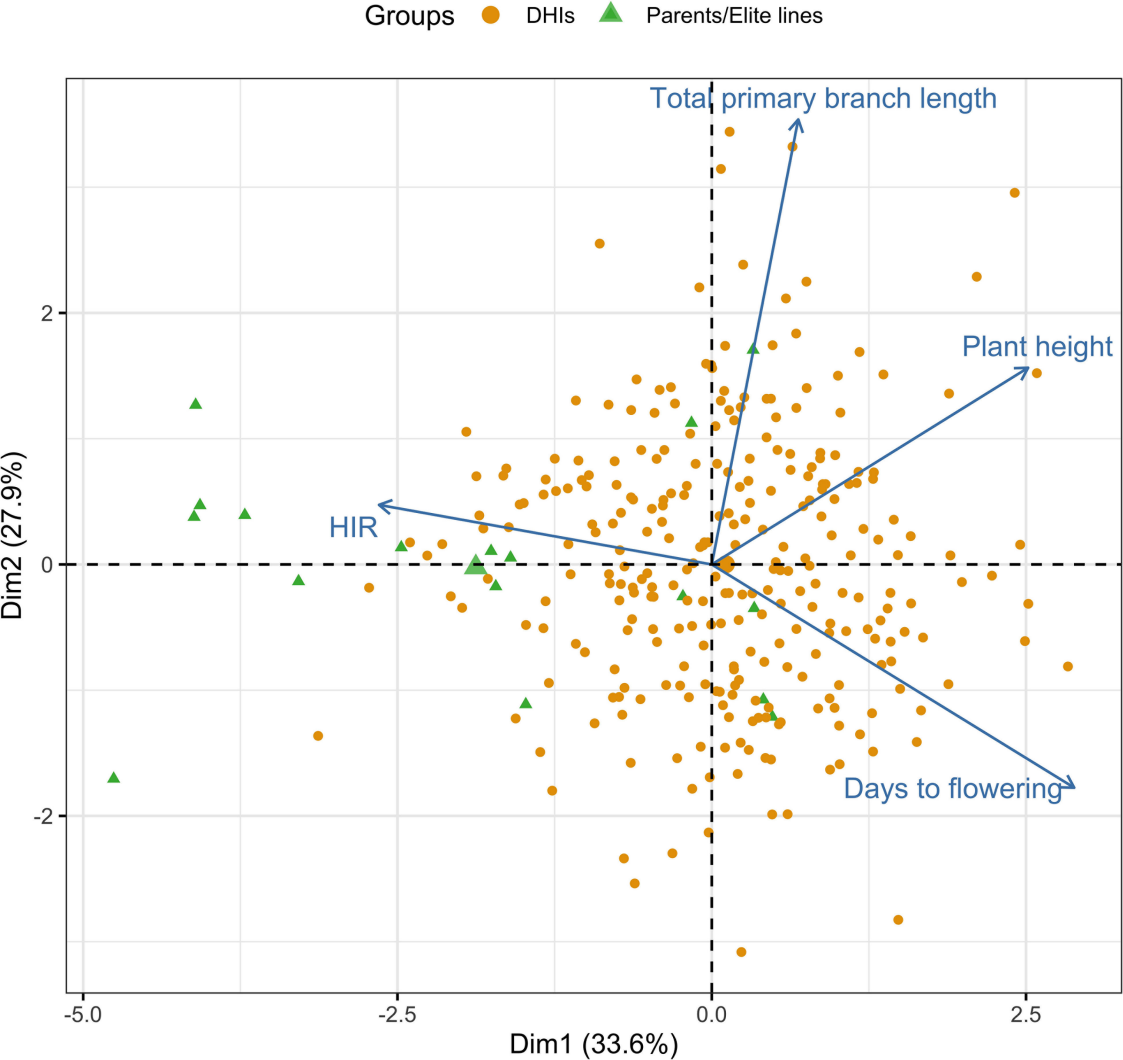
Principal component bi-plot of the four traits using the best linear unbiased estimator (BLUE) values of 283 DHI lines, nine inducer parents, and eight elite inducer inbreds in the multi-trait genomic prediction models. DHI, doubled haploid inducer.

**Figure 3:**
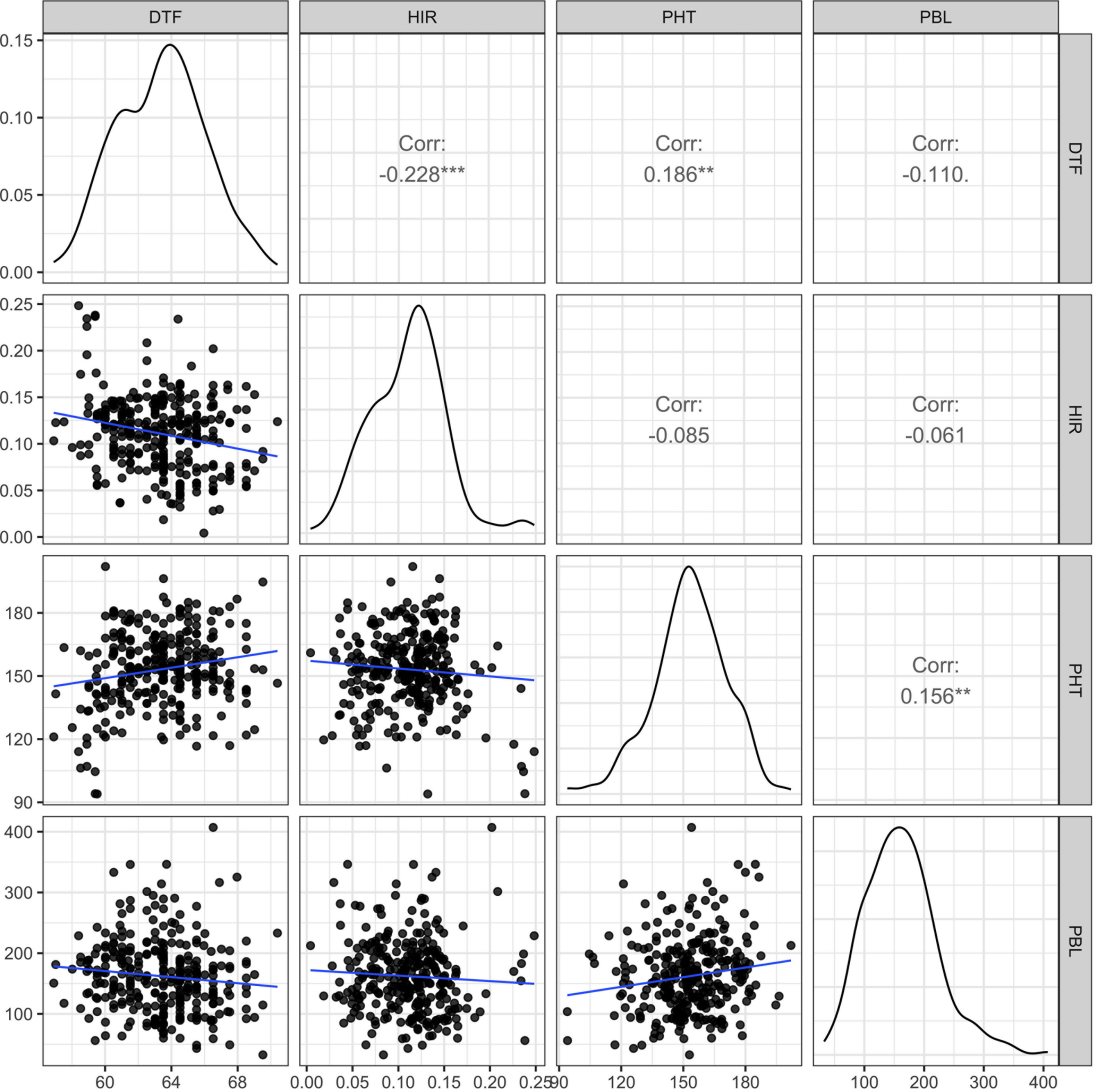
The scatter plot matrix with genotypic distributions and Pearson's correlation coefficients between the four traits of 283 DHI lines, nine inducer parents, and eight elite inducer inbreds. DTF, days to flowering; HIR, haploid induction rate (%); PHT, plant height (cm); PBL, total primary branch length (cm); DHI, doubled haploid inducer. Corr is Pearson's correlation coefficient, and **, and *** indicate values significantly different from zero at alpha levels of 0.01, and 0.001, respectively.

In the principal component analysis, the first and second PCs explained 33.6% and 27.9% of the total variation, respectively (**Figure 2**). The proportions of variance explained by the traits DTF, HIR, PHT, and PBL were 30%, 18%, 28%, and 24%, respectively (**Table 1**). PC1 was mostly explained by PHT, DTF, and HIR, and PC2 by PBL (**Figure 2**). No strong phenotypic correlations were found among the four traits studied (**Figure 3**). DTF and HIR (r = −0.228) were significantly negatively correlated (p = 0.001). DTF (r = 0.186) and PBL (r = 0.156) were significantly positively correlated with PHT (p = 0.01).

### 3.2 SNPs associated with traits in GWASs

**Figure 4:**
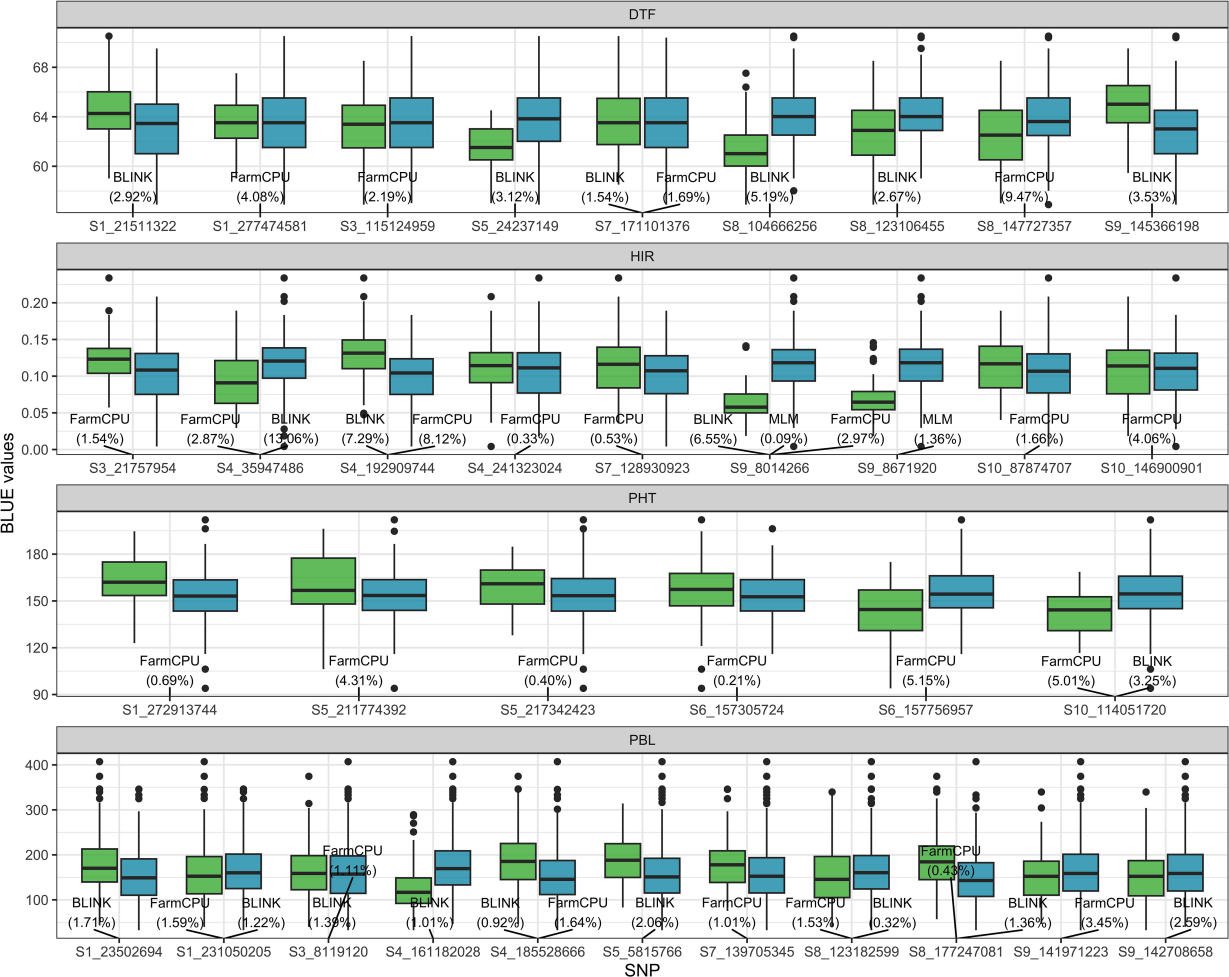
The boxplot of BLUE values of the four traits associated with SNP genotypes detected by MLM, FarmCPU, or BLINK algorithm in the GWAS panel. The value in the parentheses is the proportion of the variance explained (PVE) by detected SNP. The light green and blue boxplots represent SNP genotypes −1 (minor allele) and 1 (major allele) of the DHIs, respectively. The names of SNPs are the chromosome and position at which SNPs are located. BLUE, best linear unbiased estimator; GWAS, genome-wide association study; MLM, mixed linear model; FarmCPU, fixed and random model circulating probability unification; BLINK, Bayesian information and linkage disequilibrium iteratively nested keyway.

The SNPs detected by GWASs from the training folds during the cross-validation procedure are provided in **Supplementary Table 3**. In addition, the results of GWASs on the four traits of the training set are summarized in **Figure 4** (**Supplementary Table 4**). For DTF, six and four SNPs were detected by BLINK and FarmCPU, respectively, on chromosomes 1, 3, 5, 7, 8, and 9. Among the detected SNPs for DTF, S7_171101376 was the only one shared SNP by BLINK and FarmCPU. For HIR, three, eight, and two SNPs were detected by BLINK, FarmCPU, and MLM, respectively, on chromosomes 3, 4, 7, 9, and 10. S4_35949486 and S4_1929099744 were jointly identified by BLINK and FarmCPU, and S9_8014266 by all three models. S9_8014266 was also the most frequently detected SNP (21 out of 25 times by FarmCPU) in the CV (**Supplementary Table 3**). For PHT, one and six SNPs were detected by BLINK and FarmCPU, respectively, on chromosomes 1, 5, 6, and 10. S10_114051720S7 was the only SNP detected by both BLINK and FarmCPU. For PBL, nine and seven SNPs were detected by BLINK and FarmCPU, respectively, on chromosomes 1, 3, 4, 5, 7, 8, and 9. Five SNPs, S1_231050205, S3_8119120, S4_185528666, S8_123182599, and S8_177247081, were common among BLINK and FarmCPU. There was no SNP detected in four out of fivefold GWASs in the training set at more than 13 out of 25 times for DTF, PHT, and PBL in the CV (**Supplementary Table 3**).

### 3.3 PAs of traits by the different GP models

The PAs of the four traits in our GP models were moderate to high in the DHI population (**Table 2**). DTF and HIR had high PAs of 0.67 and 0.69 on average, respectively. PHT and PBL had moderate PAs of 0.57 and 0.51 on average, respectively. The performance of different GP models varied among traits. The standard deviation of the PA of DTF was 0.11, larger than that of the other traits. For model evaluation and comparison, the single-trait rrBLUP method was used as a baseline. For DTF, the single-trait BayesB method increased the PA by 5.6%, while the multi-trait and tree-based algorithmic models did not improve the PA. For DTF, PHT, and PBL, employing *de novo* GWAS covariates in the linear models performed poorly among the methods of GP compared with HIR. For HIR, the single-trait BayesB method increased the PA by 3.3%. Multi-trait rrBLUP and BayesB increased the PAs by 3.0% and 3.1%, respectively. The single-trait/multi-trait *de novo* GWAS_rrBLUP increased the PA by 0.3%, while multi-trait *de novo* GWAS_rrBLUP decreased the PA (−0.2%). Random Forest and xGBoost increased the PAs by 6.4% and 4.5%, respectively. For PHT, the single-trait BayesB method increased the PA by 1.2%. Nevertheless, multi-trait rrBLUP and BayesB increased the PAs by 2.3% and 3.7%, respectively. Random Forest slightly increased (2.9%), but xGBoost decreased the PA (−2.7%). For PBL, the single-trait BayesB method decreased the PA by 0.5%. The multi-trait rrBLUP did not improve the PA, while multi-trait BayesB increased the PA by 2.0%. However, Random Forest and xGBoost decreased the PA by 6.9% and 13.1%, respectively. Tree-based algorithmic models only improved the PA of HIR (at least 4.5% relative to the baseline rrBLUP model) but reduced the PA of the other three traits.

**Table 2:** The genomic prediction model performance of eight methods of the four traits in fivefold cross-validation.

Models	Methods	DTF		HIR		PHT		PBL	
		PA	RI	PA	RI	PA	RI	PA	RI
Linear	ST_rrBLUP	0.718	0.0%	0.678	0.0%	0.575	0.0%	0.549	0.0%
	ST_BayesB	0.758	5.6%	0.698	3.3%	0.581	1.2%	0.546	−0.5%
	ST_GWAS_rrBLUP	0.575	−19.9%	0.680	0.3%	0.538	−7.6%	0.481	−12.4%
	MT_rrBLUP	0.707	−1.5%	0.696	3.0%	0.586	2.3%	0.549	0.0%
	MT_BayesB	0.698	−2.8%	0.697	3.1%	0.593	3.7%	0.560	2.0%
	MT_GWAS_rrBLUP	0.425	−40.8%	0.677	−0.2%	0.540	−7.2%	0.441	−19.7%
Tree-based	Random Forest	0.711	−1.0%	0.717	6.4%	0.589	2.9%	0.511	−6.9%
	xGBoost	0.725	1.0%	0.705	4.5%	0.562	−2.7%	0.477	−13.1%
Average		0.665		0.694		0.571		0.511	
Standard deviation		0.111		0.014		0.022		0.044	

ST, single-trait; MT, multi-trait; PA, predictive ability; RI, relative improvement to the baseline ST_rrBLUP method; DTF, days to flowering; HIR, haploid induction rate (%); PHT, plant height (cm); PBL, primary branch length (cm).

**Figure 5:**
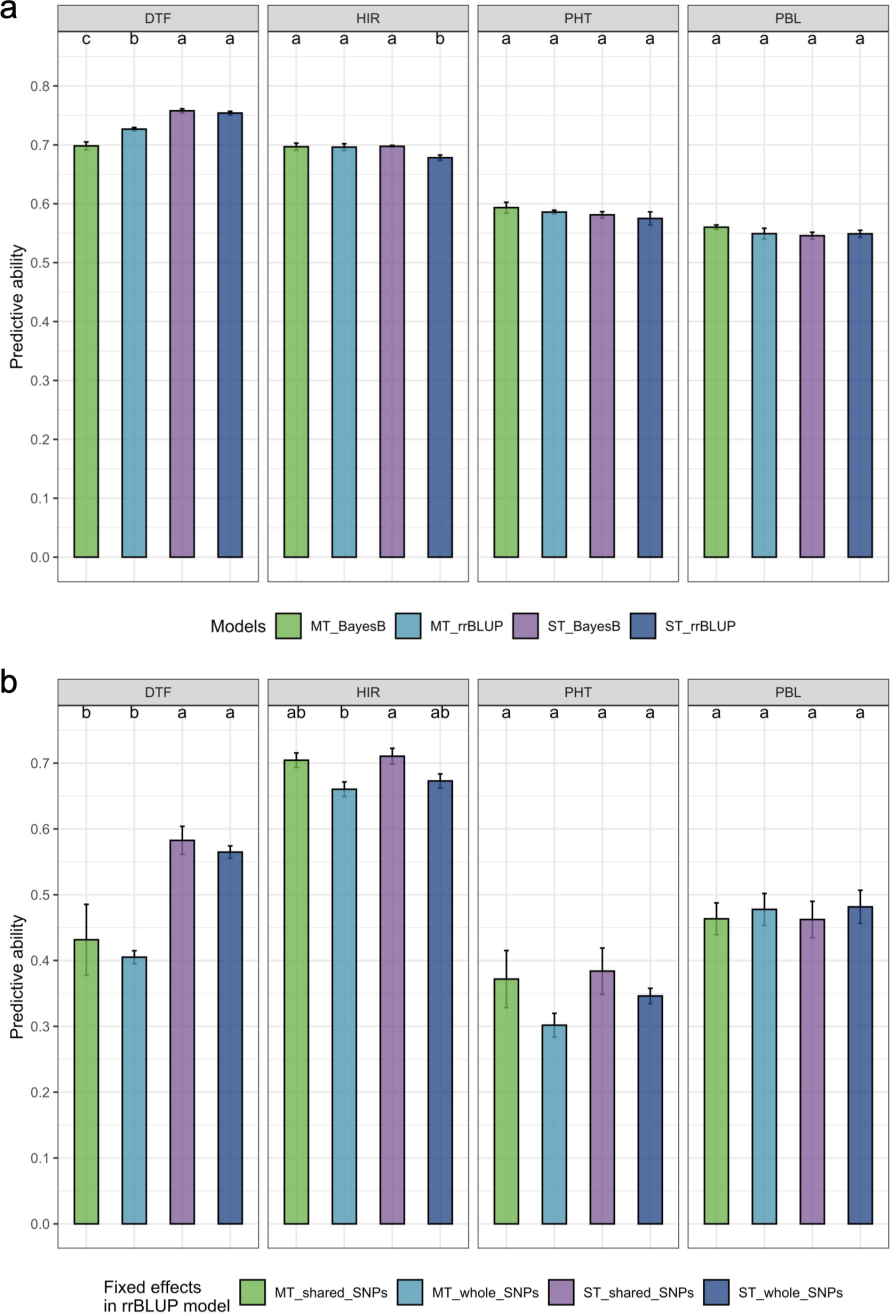
The comparison of estimated prediction ability of the four traits in the different genomic prediction models. **(a)** Comparison of single-trait/multi-trait and BayesB/rrBLUP methods. **(b)** Comparison of single-trait/multi-trait models and shared SNPs/all detected SNPs in GWAS treated as fixed factors in the rrBLUP model. DTF, days to flowering; HIR, haploid induction rate (%); PHT, plant height (cm); PBL, primary branch length (cm). The comparisons were only conducted in the split folds, which have shared SNPs detected by the three GWAS models in the cross-validation. The letters represent the groups after the LSD test at 0.05 alpha level. A common letter indicates no significant difference in predictive ability. The standard error of prediction ability in fivefold cross-validation is presented on the bar. rrBLUP, ridge regression best linear unbiased prediction; GWAS, genome-wide association study; LSD, least significant difference.

The estimated PA of the multi-trait BayesB/rrBLUP method was significantly (p = 0.05) greater than that of the single-trait rrBLUP method for HIR based on an LSD test (**Figure 5a**). In contrast, the estimated PA of the multi-trait BayesB method was significantly (p = 0.05) lower than that of the single-trait BayesB and single/multi-trait rrBLUP methods for DTF (**Figure 5a**). For PHT and PBL, single- or multi-trait BayesB/rrBLUP methods were not significantly (p = 0.05) different from each other (**Figure 5a**). SNPs chosen as fixed factors in the GP models were either shared SNPs or all SNPs detected in association analyses. The PA of treating shared SNPs as fixed factors in single- or multi-trait GWAS_rrBLUP methods was not significantly (p = 0.05) different from that of all SNPs detected, but using the shared SNPs in the model resulted in a higher PA trend than all detected SNPs for the four traits (**Figure 5b**).

### 3.4 Evaluation of GP models using MAGIC DHIs

**Figure 6:**
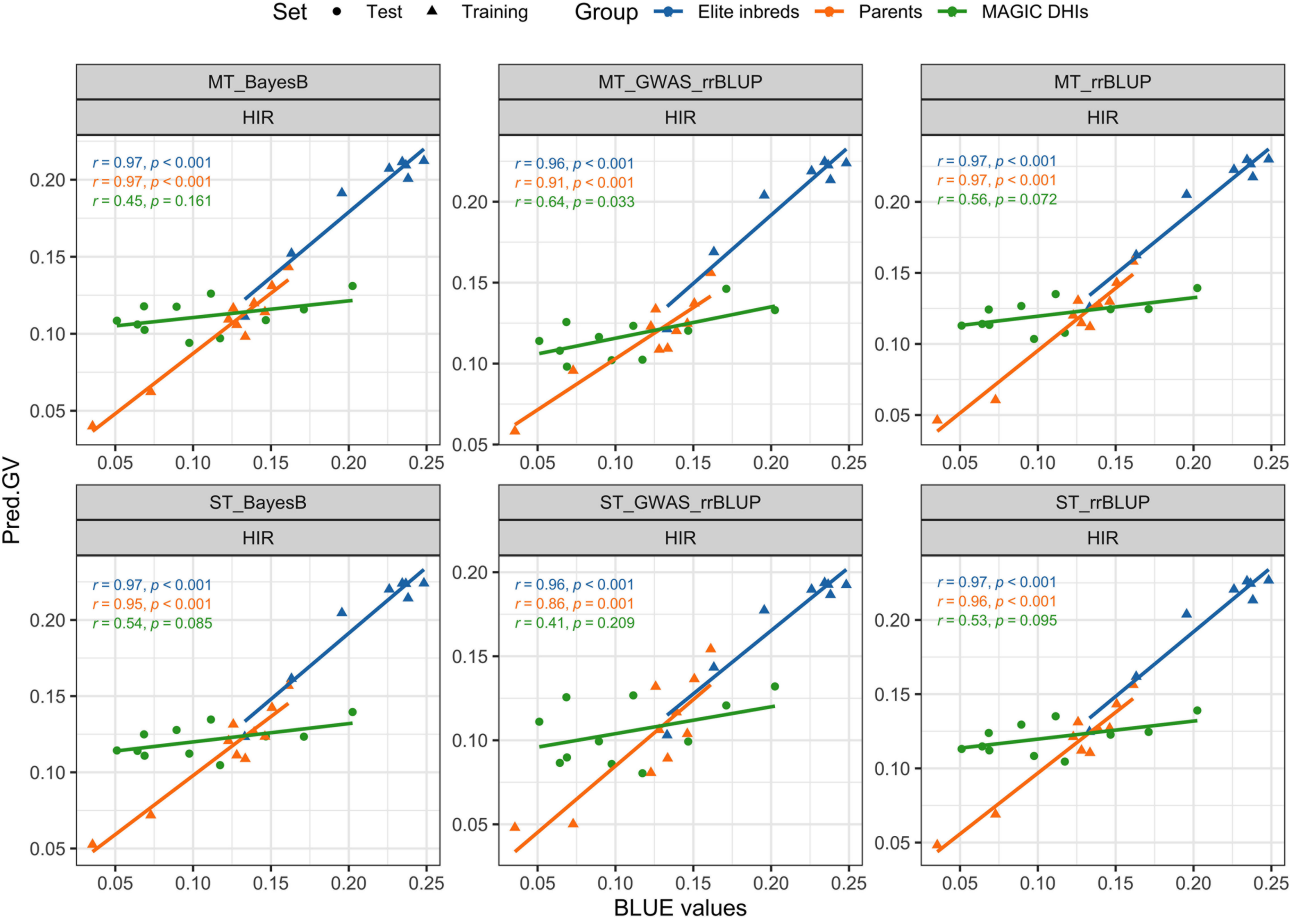
Validation of genomic prediction for HIR in the MAGIC DHI test set by different models. Pred.GV represents the predicted genotypic value from the models. r represents Pearson's correlation coefficient between Pred.GV and BLUE values, and p represents the p-value of Pearson's correlation coefficient significance tests. HIR, haploid induction rate; MAGIC, multi-parent advanced generation inter-cross; DHI, doubled haploid inducer; BLUE, best linear unbiased estimator.

The PAs of single-trait BayesB and rrBLUP methods exceeded 0.92 (p < 0.001) for DTF in MAGIC DHI populations (**Table 3**). Likewise, the multi-trait rrBLUP method showed a PA of 0.94 (p < 0.001), while the PA of multiple-trait BayesB was 0.88 (p < 0.001). The PA of the single-trait GWAS_rrBLUP method was 0.49 (p = 0.127) but increased to 0.8 (p = 0.003) when using multi-trait GWAS_rrBLUP. For the prediction of HIR in the MAGIC DHI test set, the PAs of single- or multi-trait BayesB and rrBLUP methods and single-trait GWAS_rrBLUP methods were between 0.41 and 0.56, which had no clear linear correlation relationship (p > 0.05). Nevertheless, the PA of the multi-trait GWAS_rrBLUP method for HIR (0.6) was significant (p = 0.033). Using the *de novo* GWASs (SNPs as fixed factors) improved the PA of HIR but not DTF, PHT, and PBL in the MAGIC DHIs (**Table 3**; **Figure 6**). The PAs of single-trait BayesB and rrBLUP methods for PHT were significant (p < 0.05) and exceeded 0.70. The PAs of PHT and PBL increased using the multi-trait approach in each method of the GP models in the MAGIC DHI test set (**Table 3**). Overall, multi-trait GP models improved the PA by 12% compared to single-trait GP models across four traits (**Table 3**).

**Table 3:** The predictive ability of different genomic prediction models in the MAGIC DHI test set.

Traits	Multi-trait			Single-trait		
	MT_rrBLUP	MT_BayesB	MT_GWAS_rrBLUP	ST_rrBLUP	ST_BayesB	ST_GWAS_rrBLUP
DTF	0.94 (<0.001)	0.88 (<0.001)	0.8 (0.003)	0.92 (<0.001)	0.94 (<0.001)	0.49 (0.127)
HIR	0.56 (0.085)	0.45 (0.161)	0.64 (0.033)	0.53 (0.095)	0.54 (0.085)	0.41 (0.209)
PHT	0.8 (0.001)	0.87 (0.002)	0.72 (0.029)	0.72 (0.028)	0.7 (0.036)	0.61 (0.082)
PBL	0.65 (0.081)	0.63 (0.096)	0.25 (0.547)	0.43 (0.289)	0.38 (0.347)	0.04 (0.921)
Average	0.74	0.71	0.60	0.65	0.64	0.39
		0.68			0.56	

The number in the parentheses is the p-value of Pearson's correlation coefficient significance test.

DTF, days to flowering; HIR, haploid induction rate (%); PHT, plant height (cm); PBL, primary branch length (cm); MAGIC, multi-parent advanced generation inter-cross; DHI, doubled haploid inducer.

## 4 Discussion

### 4.1 Reliability of GP experiments

GS requires a subset of genotypes from the breeding program to serve as the training set. The accuracy of GP is determined by the training population size, narrow-sense heritabilities (h^2^) of traits, and effective number of chromosome segments underlying the traits (Daetwyler et al., 2010b). Empirical and simulation studies in crops revealed that the prediction accuracy of the single-trait rrBLUP method for different heritabilities of traits reaches a plateau when the training set exceeds 250 individuals, and the number of markers is >1,000 across the genome (Asoro et al., 2011; Combs and Bernardo, 2013; Ou and Liao, 2019; Wu et al., 2023). In this study, the lines in the training set were homozygous and homogeneous DHIs from eight parents crossed in a half-diallel design and genotyped with 6,636 genome-wide SNPs. The different GP models were tested with more than 300 genotypes in the training sets by fivefold cross-validation. Adjusted phenotypic values (BLUE values) of genotypes were estimated from an unbalanced experimental design. Estimated reliabilities of the four traits reported in this study ranged from 0.65 to 0.81 (**Table 1**), and the PAs based on the single-trait rrBLUP method exceeded 0.55 (**Table 2**).

### 4.2 Performance of GP models across traits

In a previous study (Almeida et al., 2020), the PA of the single-trait rrBLUP method was evaluated by 60%/40% cross-validation in a different inducer population. DTF and HIR had estimated PA values higher than 0.72, while PHT and tassel size had PAs less than 0.72. This is consistent with our study, in which the PAs of the single-trait rrBLUP method for DTF and HIR were 0.72 and 0.68, respectively, and higher than those for PHT and PBL (0.58 and 0.55, respectively). The PA of the baseline single-trait rrBLUP method ranged from 0.55 to 0.72. Prediction accuracy, the PA divided by the square root of broad-sense heritability (*H* ^2^) (Dekkers, 2007), equal to *i* ^2^ in this study, ranged from 0.55 to 0.80. According to a simulation study of GS for maize (Heffner et al., 2010), GS with prediction accuracy >0.5 can increase genetic gain per unit time and cost by more than threefold compared to marker-assisted selection. Based on this criterion, applying the baseline single-trait rrBLUP method in GS is a promising approach for developing improved haploid inducers in maize. Nevertheless, the genetic architecture of traits influences the prediction accuracy in GP, which refers to the number of QTLs and the distribution of their effects (Daetwyler et al., 2010b). The BayesB method outperformed the rrBLUP method in the CV for DTF, HIR, and PHT by 5.6%, 3.3%, and 1.2% of PA, respectively. The inclusion of major QTLs detected in the *de novo* GWASs as a fixed factor in the rrBLUP method of GP improved the PA (Chen et al., 2023; Herter et al., 2018; Pang et al., 2021; Spindel et al., 2016). By employing the ST_GWAS_rrBLUP method in this study, the PA of HIR was improved by 0.3%, while the PAs of DTF, PHT, and PBL declined by 19.9%, 7.6%, and 12.4%, respectively. When implementing major QTLs as fixed factors in GP models for increasing PA considerably, the composition of the training population is important (Herter et al., 2018). The PA of ST_GWAS_rrBLUP in the evaluation by fivefold cross-validation was probably underestimated for the PAs of the traits in this study because GWASs were only conducted in less than 20% of the training set in the CV procedure, and the held-out set was conducted by random shuffling across multiple families of the training population rather than within families. In addition, according to the results of *de novo* GWASs in the CV (**Supplementary Table 3**), the detected SNPs were rarely consistent in the different split folds by the different models for DTF, PHT, and PBL. However, four SNPs were consistently detected for HIR in the CV, which may have resulted in an increased PA of the ST_GWAS_rrBLUP method for HIR. Furthermore, three out of the four SNPs (S4_35947486, S4_192909744, and S9_8014266) were also detected in the *de novo* GWASs of the complete training set. It can also explain the higher PA of the ST/MT_GWAS_rrBLUP method in the MAGIC DHIs. Only the tree-based models using the Random Forest and xGBoost methods exceeded the linear model rrBLUP method for the prediction of HIR. This suggests that non-linear interaction QTL effects of HIR are captured by tree-based models. Overall, the single-trait rrBLUP method had reasonable predictive ability for all traits in GS. However, multi-trait GP methods were superior for the majority of traits in our study (**Tables 2**, **3**).

### 4.3 Optimal GP models for different traits

The PA of DTF in multi-trait rrBLUP/BayesB methods declined by 1.5% and 2.8% compared to single-trait rrBLUP/BayesB methods, but the PAs of HIR, PHT, and PBL increased by at least 2% when using multi-trait rrBLUP/BayesB methods (**Table 2**). Bayesian models outperformed rrBLUP in both single-trait and multi-trait models for traits with major QTLs, with multi-trait analysis showing particular benefits. However, for polygenic traits without major QTLs, the rrBLUP and Bayesian models performed similarly, and multi-trait analysis provided a slight improvement in GP (Jia and Jannink, 2012). HIR, PHT, and PBL appear to be controlled by major QTLs in the training population, as suggested by increased PA when employing multi-trait and BayesB in the GP model (**Table 2**). In contrast, DTF did not appear to be controlled by major QTLs in our training population based on these criteria (**Figure 5a**). However, no strong major QTL SNPs were detected by GWASs for any of the four traits with PVE > 15%. The PVE of SNPs in GWASs in this study was not a straightforward indicator to determine whether traits were controlled by major QTLs in the DHI training population. The DHI training population was composed of two different parent combinations: elite by elite and elite by MHI traditional inducers (**Supplementary Table 1**). MHI had a substantially lower HIR than the other elite inducer parents. In addition, the two B73-derived and PHG83-derived elite inducers crossed with MHI had substantially smaller and larger PBL, respectively, than the other inducer parents. More extreme phenotypic differences among parents may have caused PA to be increased by the multi-trait BayesB method for HIR and PBL.

Bernardo (2014) was the first to propose that when a few major genes for a trait are known and each accounts for ≥10% of the phenotypic variance, these genes should be included as fixed effects rather than random effects in GP models. However, the amount of phenotypic variance explained by the major genes in given breeding populations is unknown before training GP models. Also, the genome-wide SNPs genotyped by sequencing for GS often do not include functional SNPs of major genes, but they could contain SNPs in LD with major QTLs. Spindel et al. (2016) were the first to propose employing *de novo* GWASs in the entire training set and treating detected SNPs as fixed factors in rrBLUP models for GS. They showed that *de novo* GWASs combined with rrBLUP outperformed the other six models in various traits of rice using the Genome-wide Efficient Mixed Model Association (GEMMA) algorithm (Zhou and Stephens, 2012) for GWASs. However, the study of Spindel et al. (2016) using the entire training set in the *de novo* GWASs overestimated PA. In our study, we used not only the single-locus algorithm MLM, which is equivalent to GEMMA, but also two other multi-locus algorithms implemented in FarmCPU and BLINK, and we also conducted GWASs only in the training folds of CV for the fixed factor selection. BLINK replaces the assumption of FarmCPU in which quantitative trait nucleotides are evenly distributed throughout the genome with LD information and replaces Restricted maximum likelihood (REML) optimization for the random effects in FarmCPU with Bayesian information criteria (Huang et al., 2019). BLINK not only improves statistical power compared to FarmCPU but also greatly reduces computing time (Huang et al., 2019). To determine the improvement of GP models by incorporating GWAS results, we used all of the SNPs detected by any of the three algorithms in this study (whole SNP). Treating whole SNPs detected in GWASs as fixed factors in the single- or multi-trait GWAS_rrBLUP model improved the PA of HIR compared to the rrBLUP model (**Table 2**). The FarmCPU and BLINK models themselves handled collinearity between SNPs in *de novo* GWASs, so they addressed the potential collinearity of fixed and random effects in the GWAS_rrBLUP model. There was no statistical difference in PA of the single- or multi-trait GWAS_rrBLUP model between using the shared SNP and the whole SNP detected in GWASs (**Figure 5b**). Due to extensive LD, SNPs detected by GWASs cannot detect single genes but detect larger genome regions. Nevertheless, the SNP S9_8014266 detected in GWASs of HIR in the training population could be associated with the *zmdmp* major gene on chromosome 9, located at the physical position 3917735–3921352 (**Figure 4**). The PVE of S9_8014266 was 0.09%, 2.97%, and 6.55% in MLM, FarmCPU, and BLINK, respectively. It did not exceed the 10% PVE threshold of major genes as fixed factors as suggested by Bernardo (2014b). Chen et al. (2023) reported that the PA was increased when the PVE of SNPs detected in GWASs exceeded 2.5% by BLINK, and the SNPs were treated as fixed terms in the rrBLUP model. The PVE of the SNP associated with HIR, S4_35947486, was 2.9% and 13.1% in FarmCPU and BLINK, respectively. This was the only SNP explaining more than 10% PVE detected by BLINK. The PVE of S4_192909744 was higher than 2.5% by both FarmCPU and BLINK. S10_146900901 explained 4.1% PVE and was only detected by FarmCPU (**Figure 4**). The prediction results in the MAGIC test set suggested that considering SNPs detected across different single- or multi-locus algorithms in *de novo* GWASs rather than using PVE as an indicator is preferable to produce *de novo* GWAS results.

### 4.4 Applications of GS in DHIs derived from multiple families and a MAGIC population

Biparental GS has been shown to outperform conventional phenotypic and marker-assisted selection by achieving a shorter breeding cycle and a higher prediction ability in simulation and empirical studies (Bernardo and Yu, 2007). Biparental population sizes can be small, and training GP models may not apply well to other biparental populations. However, numerous studies have shown that when conducting GS across multiple families or breeds in plant and animal breeding, PA increases by increasing training set size and genetic relatedness between training and selection/validation populations (De Roos et al., 2009; Habier et al., 2007; Heffner et al., 2011). In this study, the training population was composed of 23 families, and the number of individuals in each family was below 50 (minimum, 5; maximum, 48) and 14.5 on average. For each trait, the training population sizes were at least 306 genotypes (**Supplementary Table 1**). Our results suggest that developing GP models for the selection of DHIs from multiple families of different genetic source-derived inducers is feasible since the PA of the baseline GP models (rrBLUP) was 0.55–0.72 among traits (**Table 2**). A MAGIC population was used to synthesize multiple parents by a balanced mating design. Because of the increased number of recombination events in MAGIC, lines that pyramided several favorable alleles can be expected. MAGIC Recombinant inbred lines (RILs) may contain favorable gene combinations that contribute to the improvement of a trait or multiple traits across the genome (Descalsota et al., 2018; Leung et al., 2015; Zaw et al., 2019). In this study, multiple-family DHI populations derived from the nine inducer parents were used as the training set, and the MAGIC DHIs derived from the same eight out of the nine inducer parents were used as the test set. The MAGIC DHIs were interspersed among the clusters of the DHIs of the training set (**Figure 1**). The PA of the single-trait rrBLUP method of DTF, HIR, and PHT in MAGIC DHIs was moderate to high (**Table 3**). This was consistent with previous findings that synthetic populations generated by fewer than eight parents can yield high PA for GS (Schopp et al., 2017). Employing multiple-trait GP models can increase PA even further for MAGIC DHIs, while the traits in this study had medium to high estimated narrow-sense heritabilities (data not shown). The PAs of low-heritability traits (<0.2) can take advantage of leveraging the genetic correlation with high-heritability traits when using multi-trait as multiple response variables in the GP models (Gaire et al., 2022; Jia and Jannink, 2012; Shahi et al., 2022). However, the heritabilities of PHT and PBL in this study were higher than 0.2, and the predictive ability of multi-trait GP models for both traits still increased (**Table 3**).

### 4.5 GS in maternal haploid inducers

The steps for applying the GP models of this study in inducer breeding programs can be classified into two scenarios by the timing of GS. Scenario A includes the following: the training set is a subset from the target breeding population, GP models are updated by the training set, and then GS is applied to the remaining genotypes. Scenario B involves using the latest GP model and conducting GS in progenies of the breeding population, such as offspring in the next selection cycle. In scenario A, the optimal GP model is the one that has the highest PA in the evaluation of GP model performance by k-fold cross-validation in the training set (Cheng et al., 2021; Runcie and Cheng, 2019; Schrauf et al., 2021). In genotypes of inducers that did not all carry the *zmdmp* gene in the breeding population, the ST/MT_GWAS_rrBLUP increased the PA of HIR in scenario A. In addition, it also achieved high PA in HIR of MAGIC DHIs in scenario B (**Figure 6**). As a result, GS benefits from the GWAS_rrBLUP model when the training population is a mixture of *zmdmp/zmdmp* (elite inducers) and *zmDMP/zmDMP* genotypes. In scenario B, according to the results of validations of GP models in MAGIC DHIs, the multi-trait BayesB or rrBLUP methods are better than the single-trait BayesB or rrBLUP methods for our four traits. When considering PA and computation costs in GP model training together, multi-trait rrBLUP is better than the multi-trait BayesB method. When arranging the schedule of GS, it has to be considered that HIR measurements usually require extra time after harvest. However, SNP genotyping of lines can often be completed before flowering in major breeding programs. Therefore, GS could be implemented by the GP model to select candidates as parents for crossing to accelerate the breeding cycle (i.e., scenario B). Note that it is without phenotyping before and after GS. HIR is the primary target trait in maternal haploid inducer breeding. Even though DTF, PHT, and PBL were not closely correlated with HIR (**Figure 3**), the multiple-trait GP models increased the PA of HIR (**Table 2**). In the time to update GP models (i.e., scenario A), phenotyping for DTF, PHT, and PBL of inducers can be completed during the field nursery season. However, the training or updating of the GP models depends on the availability of HIR measurements as a rate-limiting step. Therefore, the streamlining of GS along with DH technology recommended in maternal haploid inducer breeding is scenario A–(scenario B)_n_–scenario A, and n is two, which refers to the number of intercrosses of MAGIC DHIs in this study (**Supplementary Figure 1**). GP models need to be updated over time because the phenotypes of lines in the target environments are determined by the composition of genes and the interactions of genes between environments.

## Data Availability

The dataset presented in this study can be found in the online repository, https://doi.org/10.25380/iastate.24527653.v1.
