# Supplementary material for: Multi-trait ridge regression BLUP with de novo GWAS improves genomic prediction for haploid induction ability of haploid inducers in maize
Source: Front Plant Sci. 2025 Aug 19;16:1614457. doi: 10.3389/fpls.2025.1614457 (PMC12401904; doi:10.3389/fpls.2025.1614457)
Supplement: Supplementary file 1 [file Table1.docx]

Supplementary Figure 1. Genomic selection workflow for haploid inducer breeding suggested from this study. The gray shading blocks represent the field seasons. n is the number of population cycles before starting to conduct GS in breeding. Scenario A is that the training set is a subset from the target DH population, GP models are updated by the training set which are the DH per se performance, and then GS is applied to the remaining DHs stored in the cold room. Scenario B is to use the latest GP model and conduct GS directly in DH progenies of the breeding population.
